# The Gynecological Work of Dr. Joseph Price1Remarks before the Minnesota Academy of Medicine, April 6, 1892.

**Published:** 1892-06

**Authors:** Geo. F. French

**Affiliations:** Minneapolis


### THE GYNECOLOGICAL WORK OF DR. JOSEPH PRICE.[1]

By GEO. F. FRENCH, A. M., M. D., Minneapolis.

Since our last meeting I have had the opportunity of observing some very remarkable surgical work and studying the methods of one of America's foremost surgeons.

Although still a young man, Dr. Joseph Price, of Philadelphia, holds a clinic for the world. While he eagerly scrutinizes and studies the work of all who have distinguished themselves in surgery, and is quick to perceive and praise excellence wherever found, his ideals are Tait and Emmet. He finds Tait's knowledge of abdominal and pelvic disease exact and positive, his skill and precision in this line of work wonderful, and says he would himself feel honored to be classed as a pupil of this model for surgeons, this teacher of teachers.

Following in the wake of Tait's teachings, Dr. Price began his work in alleys, cellars, and stable lofts, making 121 sections with but a single death, and this he ascribed to starvation. This success established his confidence in the principles expounded by Tait, and laid the foundation for his subsequent brilliant career. Skilful surgery, absolute cleanliness, irrigation, and a deftly placed drainage tube ought, he believes, to give very nearly a *nil* mortality.

Koeberle's extraperitoneal method for supra-vaginal hysterectomy in the treatment of uterine fibroids or myomata, gives, according to Dr. Price, the best results. He has had seventy-five consecutive supra-vaginal hysterectomies with four deaths, two of which were due to malignant disease; fifty vaginal hysterectomies for cancer with one death,—a record which, I am informed, has no parallel. Of abdominal sections for ectopic gestation, he has had sixty-six with two deaths, which with characteristic candor and modesty he ascribes to bad surgery; fifty-eight consecutive cases without a death. Of these, several appeared moribund when laid on the table. In some cases the operations were made precipitately, without preparation; in two, the nurses protested that the women were quite dead. In the whole series of 1,300 completed abdominal sections he has lost forty-six; the deaths have been largely in puerperal cases, appendicitis, bowel-obstruction and malignant disease. He

1. Remarks before the Minnesota Academy of Medicine, April 6, 1892.

thinks that six of the deaths were due to avoidable accidents, three to permitting his pupils to do tying, and not a few lives were lost through complications entailed by previous uncompleted operations. In one, five futile sections had been made prior to his own operation.

Dr. Price affirms that operations done in the morning exhibit a lower rate of mortality than those made in the afternoon. He improvises an operating room for each occasion, and thinks a general operating room objectionable.

I was greatly pleased to observe Dr. Price's admiration of Emmet. He said: "Tait is the master workman in all abdominal and pelvic surgery, but Emmet is my ideal surgeon in plastic work."—*Northwestern Lancet.*

---

As THE history of the influenza bacillus nears completion, so does the evidence in favor of this germ forming a spore seem more certain. This fact will explain much that we have been at a loss to account for in the occurrence and spread of past influenza epidemics. Spores of other diseases will live for years in the ground or elsewhere, and develop at any time when the circumstances are favorable. Fifty cases of control experiments and further work in the cultivation and inoculation of the Pfeiffer bacillus gave corroborative results. The bacilli disappear from the blood and sputum in from five to fourteen days, excepting in cases of lung complication, where they remain for three weeks. In the epithelial erosions, the lymphatic channels, and in the visceral organs, the germs are present in vast numbers. Inoculations on the Schniderian membrane of animals leads to fatal septic pneumonia. Thus we see that another substantiated discovery has been added to the achievements of bacteriological science.

---

CHEMOTAXIS.— *The St. Louis Medical Review* seems to take exception to this new term, because it is not found in Foster's Dictionary. This seems a very poor apology for an excuse why the term should not be used, as all words originated outside of dictionaries. The term refers to the faculty possessed by all motile bacteria of moving toward and away from certain substances which seem to attract or repel them; it is the outcome of advanced bacteriological observations and a consequent demand for a term to explain a certain condition. This faculty of *chemotaxis* is, however, not confined to bacteria; it is manifest also in plasmodia, in myxomycetes and in other unicellular organisms, hence the additional term, chemotropism, from the Greek — Τροπος — to turn.

---

Owing to its great practical importance, the time occupied in the absorption of catgut ligatures has been a subject very frequently discussed. The *process*, by softening and infiltration, then becoming imbedded in connective tissue and compressed, forming a granular detritis, undergoing liquefaction and subsequent absorption or removal by phagocytes or the wandering cells, has been understood since Flemming's substantiating investigations. The time occupied in this change which the catgut must undergo in absorption seems independent of the manner in which it is prepared, whether by VonBergmann's ether, sublimate, and alcohol method, by McEwen's chromic acid, or Lister's carbolic acid method, or by the Benkisser-Reverdin process of dehydration and deoleation, then disinfection by hot steam. These, together with Kocher's sterilization in juniper oil or Brunner's sterilization in xylol, seem *non-influential* in affecting the time which animal ligatures remain unabsorbed. Tillmanns, in his investigations, has found that up to the twenty-second day there is scarcely any change in catgut suture, and in four cases after nearly three months it remained unchanged.

Recent experiments by Hallwachs have shown that not until after six months does catgut inserted into *the bodies* of animals disappear entirely. *Repeatedly* this observation has been confirmed by operators, and not infrequently after *four weeks*' catgut, as silk, used in plastic vaginal operations, may still be seen.

There are cases on record, in which after over 1.2, 1½, or 2 *years*, catgut suture has been found in the uterus and in peritoneal cicatrices. The fear occasionally expressed that catgut ligature or suture may too rapidly disappear, therefore, seems illfounded. It persists much longer, many times, than is desired. F. J. T.

---

Bromide of Strontium in Gastric Troubles. — A great deal has recently been written in the French journals on the therapeutic value of strontium. Germain Sée has, noticing that in cases of Bright's disease digestive troubles were often very much benefited, tried the effect of bromide of strontium in different forms of gastric troubles in doses of from half a drachm a day. Different forms of dyspepsia were much benefited by its use. He suggests that the iodide of strontium may with advantage be substituted for the iodide of potassium in cases of heart disease.—*Doctors' Weekly*.

